# Supplementary figures and images for: Miro-dependent mitochondrial pool of CENP-F and its farnesylated C-terminal domain are dispensable for normal development in mice
Source: PLoS Genet. 2019 Mar 11;15(3):e1008050. doi: 10.1371/journal.pgen.1008050 (PMC6428352; doi:10.1371/journal.pgen.1008050)

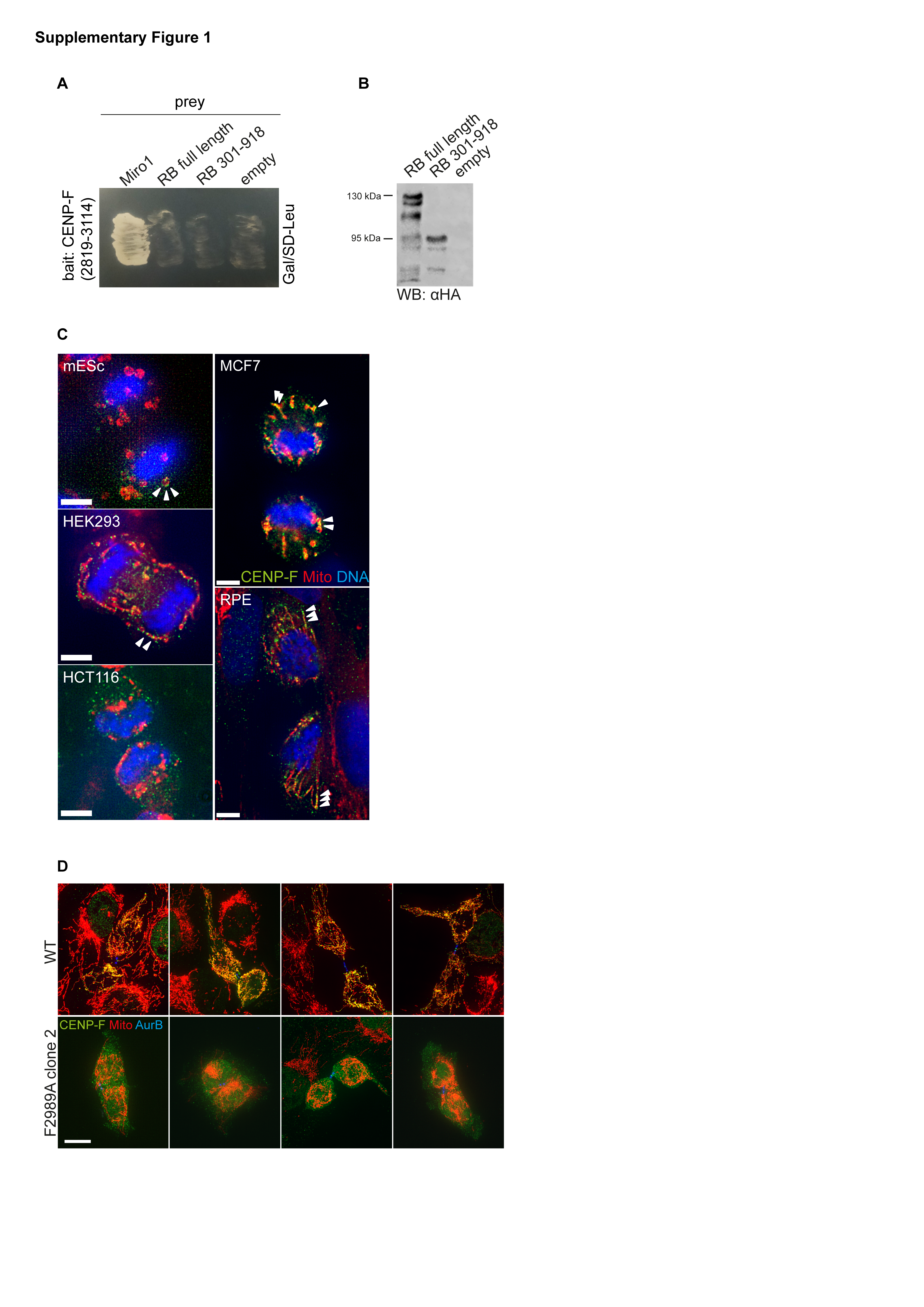

Supplement: S1 Fig — A) Yeast-two-hybrid assay between the region of CENP-F encompassing the presumed RB-binding domain and full length or fragment 301–918 of RB, fails to yield any positive signal, as assessed by the growth on medium lacking Leucine. By contrast, the same fragment of CENP-F yields robust interaction signal with Miro1. B) Western blotting of yeast extracts showing that both full-length and 301–918 fragments of RB are well expressed. Since the interaction between RB and CENP-F is deemed to be direct, and since fragments of both proteins have been successfully used in two-hybrid assays, it appears that the interaction between RB and CENP-F is weak. C) Immunofluorescence of different cell lines stained using CENP-F SCF.M or CENP-F Ab5, Aurora B, mitotracker and DAPI. D) Immunofluorescence of mtBFP-expressing cytokinetic CENP-FWT or CENP-FF2989A U2OS cells using a CENP-F antibody (Ab5). Scale bars 5 μm. (TIF) [file pgen.1008050.s001.tif]
